# Mechanistic insights into how intrapancreatic fat deposition drives the development of acute pancreatitis

**DOI:** 10.7150/ijbs.131224

**Published:** 2026-04-08

**Authors:** Xinyi Gu, Hongzhu Zhang, Wenjun Lin, Wangyang Chen, Chenyu Le, Zhicheng Huang, Qiang Liu, Jianfeng Yang

**Affiliations:** 1 Department of Geriatrics, Affiliated Hangzhou First People's Hospital Chengbei Campus, School of Medicine, Westlake University, Hangzhou 310022, China.; 2 Department of Gastroenterology, Affiliated Hangzhou First People's Hospital, School of Medicine, Westlake University, Hangzhou 310006, China.; 3 Department of Intensive Care Unit, Affiliated Hangzhou First People's Hospital Chengbei Campus, School of Medicine, Westlake University, Hangzhou 310022, China.; 4 Key Laboratory of Integrated Traditional Chinese and Western Medicine for Biliary and Pancreatic Diseases of Zhejiang Province, Hangzhou 310006, China.; 5 Hangzhou Hospital & Institute of Digestive Diseases, Hangzhou, Hangzhou 310006, China.

**Keywords:** acute pancreatitis, Intrapancreatic fat deposition, Inflammatory storm, Free fatty acids

## Abstract

Acute pancreatitis (AP) is a common emergency of the digestive system, with some cases progressing to severe acute pancreatitis (SAP), which threatens life. Intrapancreatic fat deposition (IPFD) denotes the abnormal infiltration of lipid and pathological proliferation of adipocytes in pancreatic parenchyma. It has been demonstrated to bear a close correlation with the heightened risk and progressive development of acute pancreatitis, whereas the specific molecular and cellular mechanisms underlying this association remain poorly understood. This article elaborates on the mechanisms through which IPFD exacerbates acute pancreatitis from the following four perspectives: 1. IPFD causes pancreatic cell damage via lipotoxicity, such as inducing multiple forms of cell death in pancreatic acinar cells, damaging endothelial cells to trigger microcirculatory disorders, and interfering with ductal cells leading to pancreatic duct obstruction. 2. IPFD prematurely activates pancreatic enzymes by inducing adipocyte dysfunction, thereby forming a vicious cycle. 3. IPFD amplifies inflammatory responses by interfering with endocrine regulation. 4. IPFD mediates pancreatic fibrotic dysfunction to amplify AP-associated injury. In addition, this article conducts a thorough review and raises the urgent key issues that remain to be addressed in current research on the association between IPFD and the development of AP, providing new insights for future clinical applications in the future.

## Introduction

Acute pancreatitis (AP) is one of the most common digestive system diseases, with common etiologies including bile duct stone-induced pancreatic duct obstruction, alcohol, hyperlipidemia, and post-Endoscopic Retrograde Cholangiopancreatography (ERCP) injury 1. With socio-economic development, lipid metabolism disorders caused by obesity are increasing year by year, leading to a rising proportion of AP cases induced by hyperlipidemia. Approximately 20% of AP cases can progress to severe acute pancreatitis (SAP) under certain conditions. SAP is characterized by an uncontrollable systemic inflammatory response syndrome (SIRS) and persistent multiple organ dysfunction syndrome (MODS), resulting in high mortality and various complications. Carr and colleagues 2 compared the severity of hyperlipidemic acute pancreatitis (HLAP) with AP from other causes and found a higher incidence of SAP in HLAP patients. In its pathogenesis, the aberrant activation of acinar cells during AP leads to continuous release of lipase, which can catalyze the hydrolysis of triglycerides in adipocytes, producing large amounts of unsaturated fatty acids. Furthermore, damaged adipocytes can inhibit mitochondrial complex I and complex V, ultimately exacerbating the necrotic process of acinar cells 3. This indicates that lipid metabolism plays a significant role in the progression of AP to SAP.

Intrapancreatic fat deposition (IPFD) is defined as a pathological state characterized by the abnormal infiltration and excessive accumulation of fat in pancreatic tissue as well as the abnormal proliferation of adipocytes. The intralobular and interlobular regions of the pancreas are susceptible to this condition, with consequent changes in the morphology, structure and microenvironment of pancreatic tissue 4, 5. A small amount of IPFD exists in the normal pancreas, with a fat content generally less than 6.2%, while pathological IPFD has a fat content exceeding 10.4% 4, 6, 7. Research into the prevalence of IPFD remains limited to date, and most relevant studies have focused on Asian populations 8. These investigations demonstrate that the prevalence of IPFD in the general population ranges from 16% to 35%, with non-obese individuals contributing up to 50% of cases 8-10. Furthermore, emerging evidence has revealed ethnic disparities in IPFD prevalence, with obese Latino individuals exhibiting higher pancreatic triglyceride levels compared to their obese African American counterparts 11. In contrast to other pancreatic diseases, IPFD has not garnered widespread attention from scholars and even lacks a unified professional term. It has been described as “pancreatic steatosis”, “pancreatic fat infiltration”, “fatty pancreas”, “intrapancreatic fat”, “intrapancreatic adipocytes” and “non-alcoholic fatty pancreas disease (NAFPD)”, which greatly limits research on its prevalence in the general population and ignores its potential danger to pancreatic tissue 6, 12-16. Multiple studies have confirmed that IPFD increases the risk of AP. A retrospective study suggested that patients with a history of AP episodes have higher pancreatic fat content than healthy controls 14. Meanwhile, a study found that in individuals without prior pancreatic disease, each 1% increase in pancreatic fat content was associated with a 32% increased probability of mild acute pancreatitis (MAP) episodes 17. It is noticeable that AP patients with concomitant IPFD were more likely to advance to SAP, according to retrospective research involving 409 AP patients 18. These studies suggest that IPFD plays a crucial biological role in the occurrence and progression of AP, but its specific mechanisms have not been systematically elucidated. This article primarily reviews the mechanisms by which IPFD contributes to the progression to AP.

## IPFD promotes AP development through lipotoxicity

The fat accumulated in pancreatic tissue exhibits substantial diversity in origin and form. It derives not only from the ectopic migration of hepatic fat or other visceral adipose tissue but also from the accumulation of intralobular and interlobular fat in pancreatic parenchyma and associated cellular differentiation processes, including lipid droplets in acinar cells, lipid droplets in pancreatic islets of Langerhans, and acinar cell-to-adipocyte transdifferentiation 4, 8, 19. However, regardless of whether it is visceral fat ectopically localized to the pancreas or endogenously formed lipid droplets within pancreatic tissue, both types can promote the progression of acute pancreatitis. Persistent intracellular crosstalk between lipid droplets and the endoplasmic reticulum in pancreatic acinar cells elicits mitochondrial calcium overload, which further exacerbates pancreatic acinar cell damage 4, 20. Insulin resistance (IR) and hepatic metabolic disorders caused by long-term nutritional excess lead to increased hepatic triglyceride production and release, with large amounts of triglycerides migrating and accumulating within the pancreas 21, 22. During an AP episode, pancreatic lipase is inappropriately activated. Under the action of pancreatic lipase, triglycerides are hydrolyzed into free fatty acids (FFAs) 23. The AP is exacerbated by these FFAs, which continuously accumulate within pancreatic lobules and damage pancreatic acinar cells, vascular endothelial cells, and pancreatic ductal cells.

### Pancreatic acinar cells

**Figure 1:**
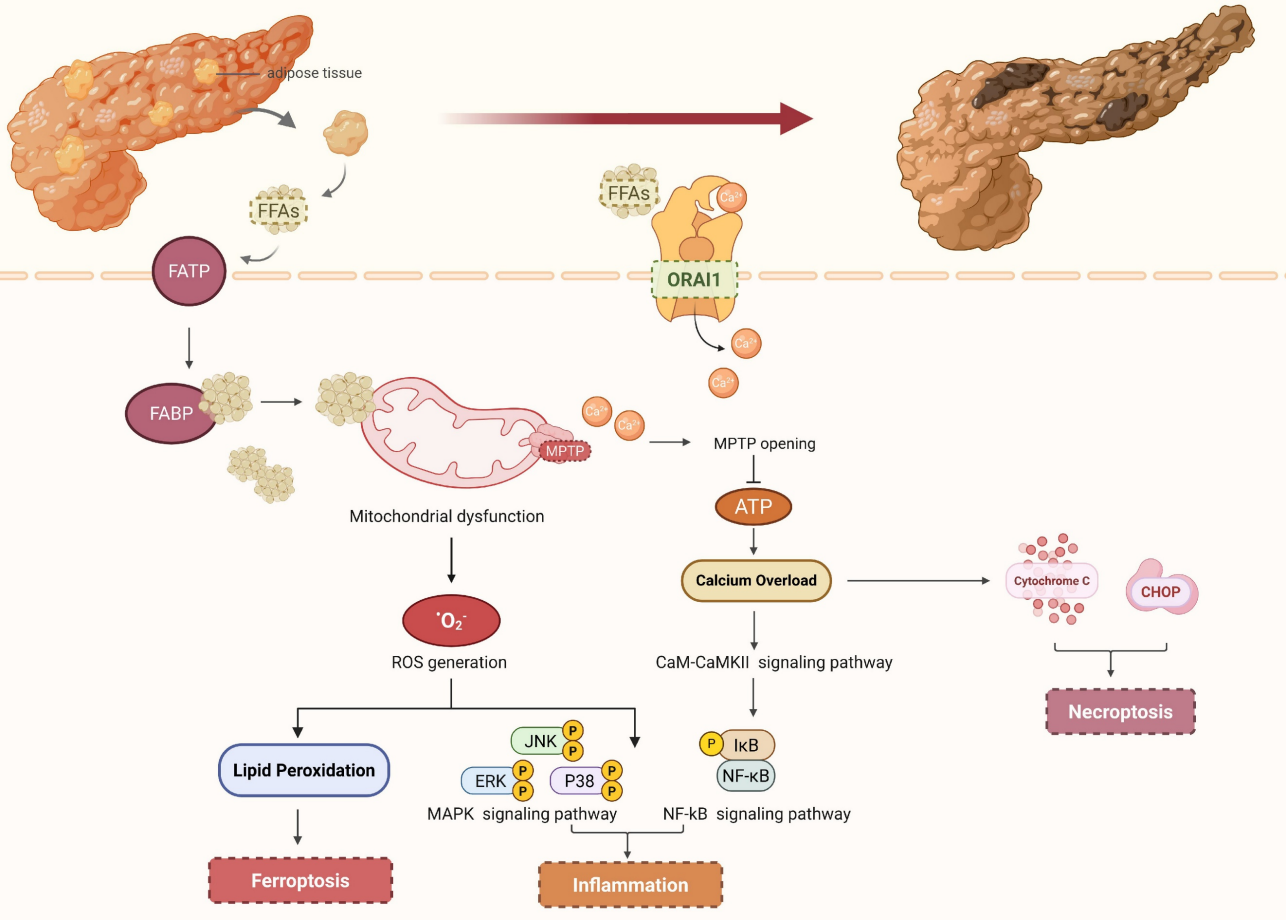
** IPFD damages pancreatic acinar cells via free fatty acids.** IPFD hydrolyzes free fatty acids, which are transported into pancreatic acinar cells via FATP and FABP on the cell surface. FFAs are subjected to mitochondrial β-oxidation, which results in the excessive generation of ROS and the subsequent activation of inflammatory signaling pathways as well as ferroptosis signaling pathways. Furthermore, high concentrations of FFAs can induce the activation of calcium channels, causing a sharp rise in intracellular Ca²⁺ levels, which triggers the opening of MPTP, disrupts mitochondrial homeostasis, impairs ATP biosynthesis, and ultimately induces calcium overload, thereby contributing to the formation of an inflammatory storm. Notably, calcium overload can also modulate the activity of downstream cell cycle regulatory proteins, thereby facilitating the necroptosis of pancreatic acinar cells. (FFAs, free fatty acids; FATP, fatty acid transporter proteins; FABP, fatty acid-binding proteins; ROS, reactive oxygen species; MPTP, mitochondrial permeability transition pores; ORAI1, calcium channel protein 1).

Pancreatic acinar cells are primarily damaged by FFAs through their binding to fatty acid transporter proteins (FATP) and fatty acid-binding proteins (FABP) on the cell surface, which interferes with intracellular lipid metabolism and leads to intracellular fatty acid accumulation 24. On one hand, high concentrations of fatty acids stimulate the opening of calcium channels on the cell membrane, causing a sharp increase in intracellular calcium ion (Ca²⁺) concentration. Subsequently, calcium channel protein 1 (ORAI1) is further activated by the high concentration of calcium ions, facilitating the transport of Ca²⁺ from outside to inside the cell and thereby maintaining high intracellular calcium levels 25. Mitochondria, as the energy metabolism center of cells, are extremely sensitive to changes in intracellular Ca²⁺ concentration. The mitochondrial permeability transition pore (MPTP) opens when the concentration of calcium ions in pancreatic acinar cells exceeds a certain threshold. This disrupts the homeostasis of the mitochondrial microenvironment and interferes with the normal electron transport chain, impeding the production of ATP and weakening the capacity to remove Ca²⁺, resulting in calcium overload 26, 27. Calcium overload is widely recognized as a pivotal event in the pathological progression from AP to SAP. Mitochondrial and endoplasmic reticulum (ER) homeostasis within pancreatic acinar cells can be directly disrupted by calcium overload, which triggers the release of cytochrome c, apoptosis-inducing factor (AIFM1), C/EBP homologous protein (CHOP), and other necroptotic mediators, thereby initiating the necroptotic signaling pathway 28. Furthermore, calcium overload regulates the calmodulin-calmodulin dependent protein kinase II (CaM-CaMKII) signaling pathway, thereby phosphorylating and activating transcription factors such as nuclear factor κB (NF-κB) and activator protein-1 (AP-1). These transcription factors enter the nucleus and regulate the gene transcriptional expression of pro-inflammatory factors such as interleukin-1β (IL-1β), interleukin-6 (IL-6), and tumor necrosis factor-α (TNF-α), triggering an "inflammatory storm" and promoting the spread of local inflammation to multiple organ systems 29, 30. On the other hand, excessive FFAs are subjected to mitochondrial β-oxidation, which results in the excessive generation of reactive oxygen species (ROS) and subsequent induction of oxidative stress-mediated cellular damage 31. As important signaling molecules, ROS can activate multiple inflammatory pathways to release numerous inflammatory factors. For instance, ROS can induce the oxidative modification of IκB kinase (IKK), which subsequently triggers the phosphorylation of IKK. This phosphorylation event facilitates the release and nuclear translocation of nuclear factor-κB (NF-κB), a transcription factor that binds to the promoter regions of target genes. The expression of the aforementioned pro-inflammatory cytokines is upregulated, thereby exacerbating the inflammatory response during pancreatitis 32. ROS can activate members of the mitogen-activated protein kinase (MAPK) family, such as extracellular signal-regulated kinases (ERK) and c-Jun N-terminal kinases (JNK). This activation modulates the activity of downstream cell cycle regulatory proteins and facilitates necroptosis of pancreatic acinar cells, ultimately contributing to the progression of AP severity 33. Additionally, when the oxidative level of ROS within pancreatic acinar cells exceeds the antioxidant capacity of Glutathione Peroxidase 4 (GPX4) and disrupts the original redox homeostasis, it can induce ferroptosis. Studies have shown that activating ferroptosis can exacerbate the progression to AP 34, 35 (Figure 1). Meanwhile, ROS can also damage vascular endothelial cells and prematurely activate pancreatic enzymes to exacerbate pancreatic injury, the mechanisms of which will be discussed later.

Dynamic interactions between lipid droplets and ER membrane contact sites (MCSs) are known to regulate lipid metabolism and protein synthesis homeostasis in pancreatic acinar cells under physiological conditions 36. In the IPFD state, an increase in acinar lipid droplet number and an abnormal expansion of their contact area with the ER trigger the continuous release of non-esterified fatty acids (NEFAs)^4^. Upon influx into the ER, these fatty acids exceed the organelle's metabolic and storage capacities, which impairs membrane fluidity and stability to a significant extent and results in a compromised ability of the ER to handle unfolded proteins 36, 37. In response to proteotoxic stress from impaired protein folding, the unfolded protein response (UPR) is initiated by the ER, and this adaptive response restores ER homeostasis through the activation of three well-characterized signaling pathways: inositol-requiring enzyme 1 (IRE1), protein kinase R-like endoplasmic reticulum kinase (PERK), and activating transcription factor 6 (ATF6) 38-40. Through these pathways, protein folding is modulated, protein synthesis is downregulated, and the degradation of misfolded proteins is promoted. However, sustained endoplasmic reticulum stress triggered by lipid droplets results in the excessive activation of the UPR. X-box binding protein 1 (XBP1) is spliced by IRE1α to form its biologically active variant, and the ATF6 pathway is activated simultaneously, which together elevate the expression of pro-inflammatory factors such as TNF-α and IL-6^38^, ^41^. Furthermore, activation of the PERK pathway induces the expression of CHOP, which triggers pancreatic acinar cell apoptosis and ultimately drives the pathological progression of AP^39^. Equally important, the ER constitutes the principal intracellular calcium pool, and maintaining the balance of calcium release and reuptake is essential for sustaining acinar cell homeostasis 42. However, FFAs released by lipid droplets open the IP3R calcium channel on the endoplasmic reticulum, which triggers cytosolic calcium overload and exacerbates the severity of AP 43, 44.

### Vascular endothelial cells

Studies have shown that pancreatic microcirculatory disturbance is a major factor causing SAP, and the two are positively correlated 45, 46. The pancreatic lobule is universally recognized as the fundamental structural and functional unit of the pancreatic microcirculatory network, which facilitates bidirectional material exchange between pancreatic parenchymal tissue and the systemic circulatory system 47. Under physiological conditions, pancreatic lobules have stable capillary perfusion 48. However, when pancreatic vasculature is damaged, arteriolar spasm increases pre-capillary resistance, lowers microcirculatory perfusion pressure, leads to insufficient capillary bed perfusion, and worsens damage to acinar cells. Simultaneously, intense arteriolar constriction slows blood flow and promotes thrombus formation. When thrombi obstruct capillaries and venules, they hinder blood return, causing microcirculatory stasis. This leads to an inadequate supply of oxygen and nutrients to pancreatic tissue, while metabolic waste cannot be cleared promptly. Consequently, pancreatic ischemia and hypoxia occur, disrupting the normal metabolism and function of pancreatic cells, and ultimately accelerating the progression from AP to SAP 49.

**Figure 2:**
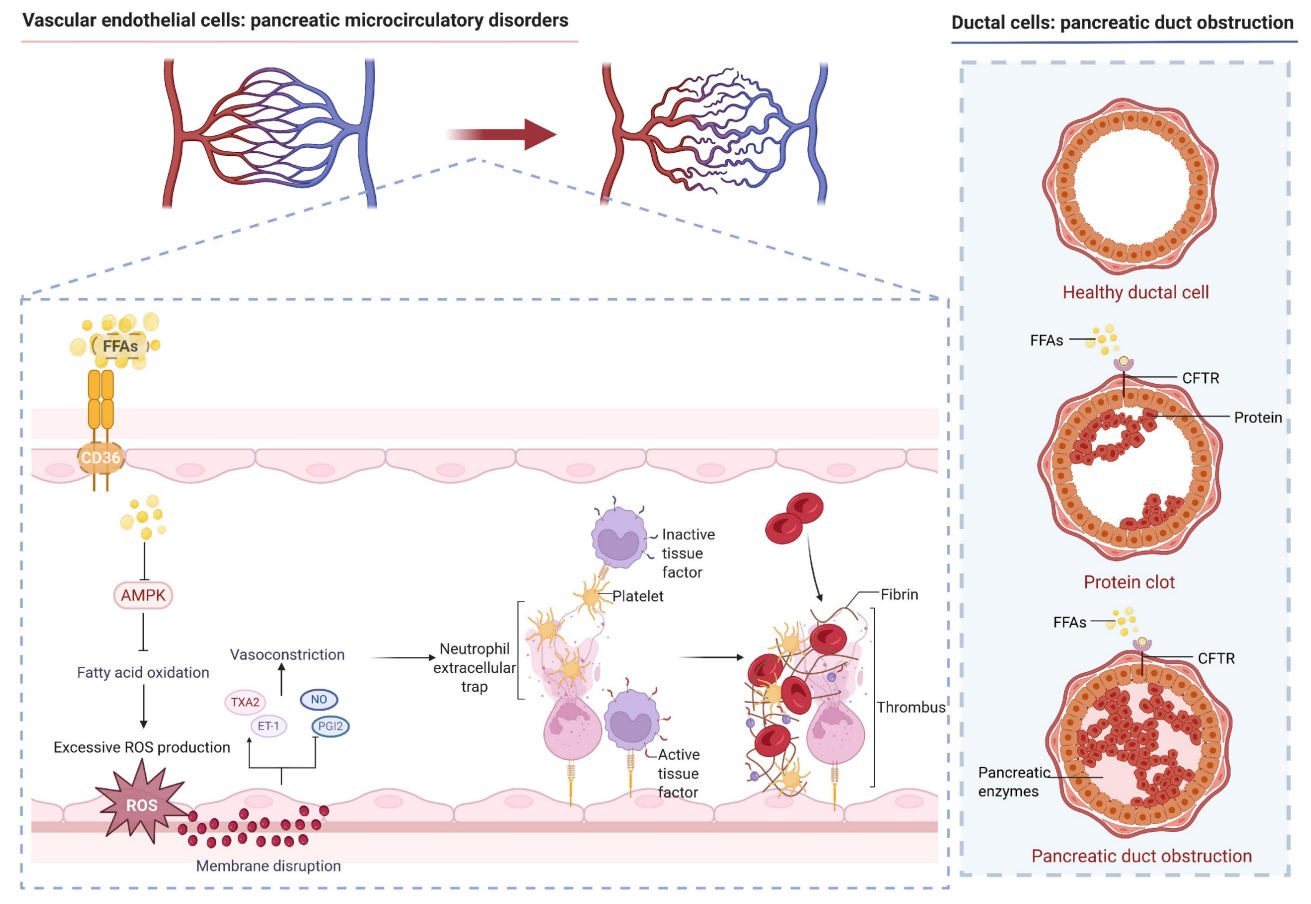
** IPFD damages vascular endothelial cells and ductal cells via free fatty acids.** FFAs bind to CD36 on the surface of vascular endothelial cells, inhibit the AMPK pathway, produce large amounts of ROS, and damage membrane lipids. Damaged blood vessels contract strongly, leading to insufficient pancreatic microcirculatory perfusion, and recruit immune cells and platelets to aggregate and form thrombi, resulting in pancreatic ischemic necrosis. FFAs impair the function of CFTR on the surface of ductal cells by affecting the absorption of fat-soluble vitamins, inducing the formation of protein clots in the duct and preventing the smooth excretion of pancreatic juice, ultimately causing pancreatic duct obstruction. (FFAs, free fatty acids; ROS, reactive oxygen species; CFTR, cystic fibrosis transmembrane conductance regulator protein).

Vascular endothelial cell damage is the starting point of pancreatic microcirculatory disturbance. FFAs can bind to Cluster of Differentiation 36 (CD36) on the surface of vascular endothelial cells, inhibit the Adenosine Monophosphate-Activated Protein Kinase (AMPK) signaling pathway, and induce oxidative stress, producing large amounts of ROS 27, 50, 51. ROS can directly act on membrane lipids, impair the integrity of vascular endothelial cells, enhance capillary permeability, induce the extracellular leakage of intracellular substances, and ultimately lead to microcirculatory disturbances. After endothelial cell injury, synthesis of vasodilatory substances such as nitric oxide (NO) and prostacyclin (PGI₂) decreases, while secretion of vasoconstrictive substances like endothelin-1 (ET-1) and thromboxane A₂ (TXA₂) increases, causing intense vasoconstriction and insufficient pancreatic microcirculatory perfusion 52. Furthermore, damage to endothelial cell integrity leads to the attraction and aggregation of platelets at the injured site, forming microthrombi. Concurrently, pancreatic microcirculatory disturbance induces a hypercoagulable state, which promotes the activation of coagulation factor VII and tissue factor (TF). This activation triggers thrombin generation and the gradual formation of a fibrin network, ultimately resulting in thrombosis that obstructs pancreatic vascular supply and causes pancreatic ischemic necrosis 53, 54. Moreover, after endothelial cell damage, cell swelling and detachment gradually accumulate within blood vessels, which can stimulate the migration of pro-inflammatory factors and chemokines, such as high mobility group protein B1 (HMGB-1) and vascular endothelial growth factor (VEGF), mediating macrophage and monocyte infiltration and exacerbating the inflammatory response 55, 56 (Figure 2).

### Ductal cells

To date, no substantial research has demonstrated that IPFD directly impairs the morphology and function of pancreatic ducts. Although no direct structural or functional association exists between IPFD and pancreatic ducts, FFAs derived from lipid hydrolysis in IPFD can induce perturbations in the pancreatic microenvironment through lipotoxicity and physical obstruction, which in turn contributes to the exacerbation of acute pancreatitis. In terms of chemical toxicity, the mechanism of FFAs is similar to that in pancreatic acinar cells, which can bind to the cystic fibrosis transmembrane conductance regulator protein (CFTR) on ductal cell surfaces, inducing calcium overload and interfering with normal energy metabolism 57, 58. Regarding physical obstruction, CFTR in ductal epithelial cells secretes abundant alkaline fluid to maintain the pancreatic alkaline environment, effectively inhibiting protein precipitation in pancreatic juice 59. When FFAs impair the absorption of fat-soluble vitamins and induce CFTR dysfunction, pancreatic juice secretion is reduced and intraductal pH declines. These changes promote the formation of protein clots within the pancreatic ducts, which obstruct the ductal lumen, elevate intraductal pressure, and ultimately result in pancreatic parenchymal necrosis 60. Simultaneously, digestive enzymes in pancreatic juice cannot be excreted smoothly and are activated abnormally, leading to autodigestion of the pancreas and thereby accelerating the progression of AP.

Pancreatic ductal cell damage is closely related to the occurrence and development of AP. Studies have shown that some HLAP patients exhibit varying degrees of pancreatic duct dilation or obstruction on imaging 54. Impaired pancreatic juice excretion and abnormal activation of digestive enzymes due to ductal cell damage are important initiating factors for pancreatitis 23. With the progression of damage and exacerbation of the inflammatory response, pancreatic tissue undergoes pathological changes such as edema, hemorrhage, and necrosis. The inflammation can also spread to surrounding tissues, triggering SIRS and MODS, and ultimately threatening the patient's life 40. Meanwhile, Viktória Venglovecz *et al*. 61 found that early use of the CFTR potentiator Orkambi to improve pancreatic duct function can effectively reduce AP severity (Figure 2).

### IPFD remodels the pancreatic microenvironment to induce abnormal pancreatic enzyme activation

Inappropriate activation of pancreatic enzymes constitutes a key mechanism in the pathogenesis of acute pancreatitis 23. Under physiological conditions, pancreatic enzymes secreted by acinar cells exist as inactive zymogens. Under the influence of predisposing factors like alcohol or pancreatic duct obstruction, lysosome rupture releases cathepsin B (CTSB), directly activating trypsinogen. Activated trypsin further activates other zymogens, such as proelastase, prophospholipase, etc. Under the collective action of these enzymes, pancreatic tissue is digested, causing AP 62, 63. IPFD not only exacerbates pancreatic injury by inducing ductal cell damage via lipotoxicity, thereby indirectly promoting pancreatic enzyme activation, but also directly modulates the pancreatic enzyme activation cascade to participate in the progression to AP.

During the progression of IPFD, adipocyte dysfunction is central to the formation of the inflammatory microenvironment. Leptin, secreted by adipocytes, is essentially a peptide hormone widely involved in the regulation of the body's immune response. Leptin exhibits potent chemotactic activity within the immune regulatory network, induces the directional recruitment of peripheral blood monocytes and tissue macrophages to adipose tissue via concentration gradient signals, causing inflammation and further inducing pro-inflammatory cytokine expression 64, 65. Ruma G Singh *et al*. 66 found that the levels of leptin and TNF-α are significantly increased in AP patients with comorbid IPFD. TNF-α, a key pro-inflammatory cytokine in the pathogenesis of AP, can bind to tumor necrosis factor receptor 1 (TNFR1) expressed on the membranes of pancreatic acinar cells. Through this binding, intracellular MAPK signaling pathways are activated, and calcium homeostasis imbalance is induced within acinar cells, thereby reducing the activation threshold for the conversion of trypsinogen to trypsin. Simultaneously, TNF-α can upregulate the expression level of trypsinogen activation peptide (TAP) within acinar cells and accelerate the trypsinogen activation process via autocatalysis 23, 40. Furthermore, excessive accumulation of adipocytes within the pancreas can compress pancreatic ducts, leading to increased intraluminal pressure, stenosis, or even obstruction, which subsequently reduces pancreatic blood perfusion. Under ischemic and hypoxic conditions, ATP production in pancreatic acinar cells declines, resulting in decreased lysosomal membrane stability. This facilitates the release of CTSB, which co-localizes with trypsinogen and promotes its activation through catalysis 67. Another critical factor is pancreatic duct compression, which impedes unimpeded pancreatic juice excretion and heightens the risk of autoactivation of protease zymogens within the pancreatic juice 68.

**Figure 3:**
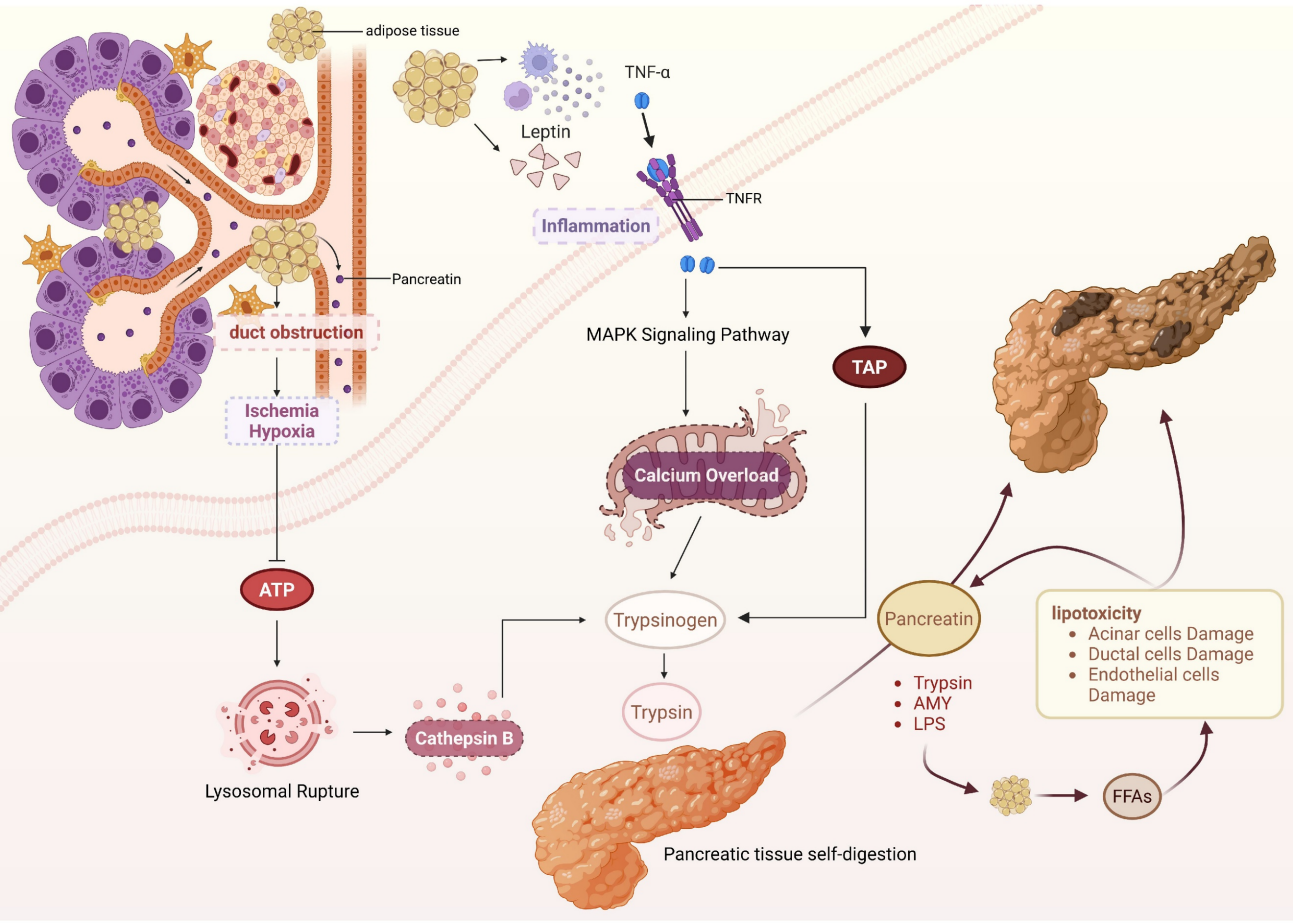
IPFD-induced pancreatic enzyme abnormal activation and vicious cycle formation. IPFD obstructs the pancreatic duct, causing pancreatic tissue ischemia and hypoxia, which reduces ATP production, induces lysosome rupture, releases cathepsin B, and directly activates trypsin. Additionally, adipocytes in IPFD trigger inflammation and activate the MAPK signaling pathway, which results in calcium overload and further contributes to the abnormal activation of pancreatic digestive enzymes. During autodigestion of pancreatic tissue induced by activated pancreatic enzymes, IPFD is hydrolyzed to release FFAs. FFAs can directly damage pancreatic tissue through lipotoxicity and further activate pancreatic enzymes by damaging pancreatic ductal cells, forming a vicious cycle and ultimately leading to AP severity. (TAP, trypsinogen activation peptide; AMY, amylase; LPS, Lipase).

IPFD-induced abnormal pancreatic enzyme activation is not a unidirectional pathological process. Activated pancreatic enzymes can regulate lipid metabolism to generate toxic mediators, forming a vicious cycle that further amplifies pancreatic damage and promotes progression to SAP. Pancreatic lipase serves as the central enzyme mediating lipid metabolism disorders. Under physiological conditions, it enters the duodenum as a zymogen and is activated by bile acids, hydrolyzing dietary triglycerides into monoglycerides and FFAs for energy. However, in the pathological state of pancreatitis, activated pancreatic lipase can be released into the intra- and interlobular spaces of the pancreas through damaged acinar cells, targeting IPFD regions and triggering massive fat hydrolysis 69. Excessive fatty acids, especially unsaturated fatty acids, enter the mitochondria of acinar cells, where mitochondrial complex activity is inhibited and large amounts of ROS are generated, ultimately resulting in acinar cell necrosis. Additionally, FFAs can accumulate in the endoplasmic reticulum of pancreatic acinar cells, which impairs protein folding function, induces pancreatic acinar cell death, and further exacerbates pancreatic tissue damage 70, 71. Researchers injected pancreatic lipase into mice with visceral fat and observed a significant decrease in their 24-hour survival rate, whereas this effect was reversed by administration of the broad-spectrum lipase inhibitor orlistat. This indirectly demonstrates that pancreatic enzymes can regulate lipid metabolism to exacerbate AP severity 69 (Figure 3).

### IPFD exacerbates AP development via dysregulation of pancreatic endocrine function

Pancreatic endocrine cells are mainly concentrated in islets, which serve as the endocrine functional units of the pancreas. Islets secrete various hormones such as insulin, glucagon, and somatostatin, and are responsible for regulating metabolic homeostasis. These hormones can modulate systemic glucose and lipid metabolism through paracrine, autocrine, and endocrine mechanisms, and are also involved in the regulation of inflammatory responses 72. Studies have shown that IPFD can disrupt pancreatic endocrine function through multiple pathways: excessive IPFD compresses islet structures and destroys islet integrity; FFAs released by adipocytes damage the cell membrane structure of endocrine cells through oxidative stress. Furthermore, IPFD disrupts the synthesis and signaling pathways of endocrine hormones, leading to hormonal imbalances, which amplifies inflammatory responses and increases pancreatic injury 73 (Table 1).

Insulin, as one of the important endogenous anti-inflammatory hormones, has been extensively studied for its anti-inflammatory mechanisms. NF-κB, a central transcriptional factor in inflammatory responses, promotes the transcription of multiple pro-inflammatory factors. Insulin binds to insulin receptors on the surface of target cells, inhibits IκB kinase activity, thereby blocking activation of the NF-κB signaling pathway and reducing the release of pro-inflammatory factors 74. Furthermore, insulin can activate the signal transducer and activator of transcription 3 (STAT3) signaling pathway, promote the expression of anti-inflammatory factors such as interleukin-10 (IL-10) and transforming growth factor-β (TGF-β), which further enhances the body's anti-inflammatory responses 75. Lipid droplets represent a key component of IPFD, and their excessive accumulation can impair the structural integrity of insulin granules and diminish insulin biological activity, consequently compromising pancreatic β-cell function 76. At the same time, studies have shown that IPFD is closely associated with insulin resistance (IR), a relationship that may be linked to lipid peroxidation and chronic inflammation 77, 78. IR markedly impairs the anti-inflammatory effects of insulin, which results in the accumulation of pro-inflammatory factors and inflammatory cells in pancreatic parenchymal tissue and further exacerbates pancreatic tissue damage. At the same time, IR suppresses the synthesis and release of NO while promoting the release of ET-1. This pathological imbalance reduces pancreatic blood perfusion, induces an ischemic and hypoxic microenvironment, and further aggravates pancreatic parenchymal injury 79. Clinical research data also corroborate this finding: a multicenter randomized controlled trial demonstrated that early insulin administration can decelerate the progression of HLAP 80.

Glucagon, the primary antagonistic hormone to insulin, is secreted by pancreatic alpha cells. It elevates peripheral blood glucose levels by promoting hepatic glycogenolysis and gluconeogenesis, while also participating in the regulation of lipolysis and inflammatory responses 81, 82. In the setting of IPFD, excessive glucagon secretion creates a hyperglycemic microenvironment that aggravates AP development. IPFD can induce elevated FFA levels, which activate G protein-coupled receptor 40 (GPR40) on alpha cell surfaces. This triggers intracellular Ca²⁺ influx, activates CaMKII, and thereby promotes glucagon synthesis and secretion 83. Furthermore, FFAs can inhibit the AMPK signaling pathway in pancreatic alpha cells. This inhibition relieves the suppressive effect of AMPK on the glucagon transcription factor Forkhead Box Protein O1 (FoxO1), which in turn enhances glucagon gene transcription 84. Multiple studies have reported that hyperglycemia may serve as an independent risk factor for both disease progression to AP and poor prognosis 85, 86. This may be related to the fact that hyperglycemia activates the Notch signaling pathway during AP injury and regulates the polarization of macrophages to the M1 phenotype, thereby intensifying the inflammatory response 87. Other studies indicate that hyperglycemic state can activate the NLRP3 inflammasome, amplify the inflammatory response and promote the progression of AP 88.

Adipose tissue not only serves energy storage and metabolic functions but also possesses certain endocrine capabilities. Correspondingly, adipocytes within IPFD can also secrete various hormones, among which leptin and adiponectin are key hormones regulating immune metabolism. Leptin has been discussed above and will not be reiterated here. Adiponectin possesses oligomerization properties within the body, typically forming trimers, hexamers, and high-molecular-weight multimers. Among these, high-molecular-weight multimers exhibit the highest affinity for target cell membrane receptors and serve as the primary form responsible for its anti-inflammatory effects 89. The predominant receptor expressed on pancreatic tissue is adiponectin receptor 1 (AdipoR1), which mediates the anti-inflammatory actions of adiponectin and enhances insulin sensitivity. In normal pancreatic tissue, specific binding to AdipoR1 on the membranes of pancreatic acinar cells is exerted by adiponectin. Through this binding, the AMPK signaling pathway is activated, lipid peroxidation and its downstream associated inflammatory factors are suppressed, and oxidative stress as well as inflammatory damage to pancreatic acinar cells are ultimately mitigated 50. Furthermore, the phosphorylation of insulin receptor substrate is promoted by adiponectin, which in turn enhances insulin action and attenuates the development of IR 90. However, in the setting of IPFD, adipocytes undergo morphological alterations and functional dysregulation, which induce endoplasmic reticulum stress (ERS) and activate the unfolded protein response (UPR). These two pathological processes impair the proper folding and processing of adiponectin precursor protein, and they ultimately bring about a marked reduction in adiponectin synthesis and secretion 91, 92. Relevant animal experiments have demonstrated that administration of recombinant adiponectin in AP mice significantly suppresses the inflammatory response and ameliorates AP 93.

Additionally, gastrointestinal hormones have received increasing attention in recent years. Among them, Ghrelin is a polypeptide hormone mainly secreted by gastric X/A cells and pancreatic islet cells, primarily regulating appetite and nutrient sensing 94, 95. Studies have shown that Ghrelin can promote the proliferation of preadipocytes and their differentiation into mature adipocytes, closely related to severe obesity seen in Prader-Willi Syndrome 96. Clinical studies have found that there is a significant positive correlation between ghrelin levels and IPFD during AP. Meanwhile, ghrelin can induce adipocytes to release FFAs and increase the risk of ectopic fat deposition 97, 98. Although Ghrelin is considered an anti-inflammatory factor, multiple clinical studies have shown that serum Ghrelin levels are significantly higher in SAP patients than in AP patients, which may be related to Ghrelin inducing pancreatic enzyme activation, but the specific mechanism remains unclear 99-102.

### IPFD mediates pancreatic fibrotic dysfunction to amplify AP-associated injury

Pancreatic fibrosis is a pathological reparative response triggered by the pancreas under conditions of sustained, recurrent injurious stimuli, and its core process is dominated by the activation of pancreatic stellate cells (PSCs). Upon receiving injurious signals, PSCs rapidly activate and differentiate into myofibroblasts, which synthesize and secrete abundant amounts of extracellular matrix (ECM) components, including type I and III collagen, fibronectin, laminin among others. These ECM components progressively deposit and replace normal pancreatic parenchyma, which ultimately gives rise to diffuse or focal fibrotic alterations 103, 104.

During an episode of AP, damaged pancreatic acinar cells release numerous pro-inflammatory cytokines and chemokines. These mediators recruit a substantial influx of inflammatory cells, such as neutrophils and macrophages, into pancreatic tissue and trigger an inflammatory storm within the local microenvironment. Within fibrotic regions of the pancreas, a physical barrier can be formed by extensively cross-linked collagen fibers, which thereby spatially restricts the dissemination of pro-inflammatory mediators to adjacent normal pancreatic and peripancreatic tissues. This helps mitigate secondary damage to intact acinar cells by inflammatory factors and prevents further expansion of the inflammatory zone 105. Additionally, the dense collagen network formed by pancreatic fibrosis can improve the damage resistance of pancreatic tissue. During the progression of AP, pancreatic acinar cells undergo edema, ischemia, and necrosis. Necrotic pancreatic tissue and activated enzymes infiltrate the peripancreatic space, potentially causing infectious pancreatic necrosis, pancreatic abscesses, and other severe complications. However, the fibrotic collagen network can counteract the tissue expansion pressure induced by pancreatic edema and isolate necrotic debris and pancreatic enzymes from healthy pancreatic tissue 106. In addition, cilia serve as mechanosensors of pancreatic ductal cells, with the capacity to perceive pancreatic fluid flow. The absence of Chibby1 (Cby1) impairs the flow-sensing function of cilia in pancreatic ductal cells and impairs pancreatic fluid excretion, resulting in pancreatic duct obstruction and a notable rise in the probability of abnormal pancreatic enzyme activation. This renders the pancreas more susceptible to the development of AP and causes more severe pathological damage after the disease initiates 107. Consequently, a reduction in the extent of pancreatic tissue fibrosis may exacerbate the progression of AP. Pancreatic fat deposition occurs in multiple sites, with the pancreatic head being the most vulnerable 108, 109. Microscopically, adipose tissue accumulation is detectable in both the intralobular regions and interlobular connective tissue of the pancreas. Specifically, intralobular fat deposition exerts a predominant impact on pancreatic parenchymal cells, such as acinar cells and endocrine cells 13. To date, the majority of studies have centered on intralobular fat deposition and its pathogenic role in pancreatic tissue injury via mechanisms including lipotoxicity and oxidative stress. Nevertheless, the clinical and pathological significance of interlobular fat deposition warrants equal attention. Studies indicate that normal visceral adipose tissue harbors abundant tissue-resident macrophages (TRMs), which can activate fibroblasts through the platelet-derived growth factor-platelet-derived growth factor receptor (PDGF-PDGFR) signaling pathway to facilitate repair of damaged pancreatic tissue. In contrast, excessive visceral fat accumulation is associated with a shift in the macrophage population, where TRMs are largely replaced by monocyte-derived C-C chemokine receptor type 2 positive (CCR2⁺) macrophages 110. This alteration attenuates the fibrotic response and may thereby aggravate the severity of AP 111, 112. Furthermore, leptin, an adipocyte-derived hormone, has been shown to reduce fibrosis in visceral tissue through mechanisms involving fatty acid oxidation 113, 114. To a certain extent, the reduction of pancreatic tissue fibrosis accelerates the progression of AP.

Conversely, other evidence indicates that FFAs derived from the hydrolysis of IPFD are capable of promoting pancreatic tissue fibrosis under specific pathological conditions. The homeostatic equilibrium of the extracellular matrix (ECM) within the pancreas is maintained by a balance between its synthesis and degradation. Matrix metalloproteinases (MMPs), a family of proteases that specifically degrade various ECM components, play a crucial role in this process. Their proteolytic activity is tightly regulated by tissue inhibitors of metalloproteinases (TIMPs), ensuring dynamic ECM stability 115. FFAs derived from IPFD hydrolysis can upregulate TIMP1 expression, leading to further inhibition of MMP activity. This disruption of the proteolytic balance results in the accumulation of undegraded ECM and ultimately promotes fibrosis 116. Pancreatic tissue fibrosis results in the replacement of normal pancreatic parenchyma with fibrotic scar tissue, which impairs the function of pancreatic acinar cells, facilitates adipocyte infiltration and proliferation within the pancreatic interstitium, and thereby exacerbates the development of IPFD 117. Through mechanisms including lipotoxicity and the induction of inappropriate pancreatic enzyme activation, IPFD elevates the risk of AP and aggravates its pathological severity. Recurrent episodes of AP further exacerbate pancreatic fat accumulation and fibrotic progression, driving the transition of acute pancreatitis to chronic pancreatitis and ultimately leading to the progressive loss of pancreatic exocrine and endocrine function 16. To date, there is no definitive consensus on the mechanisms by which IPFD modulates pancreatic fibrogenic dysfunction to drive the pathogenesis of AP. Although preliminary investigations into the relationship between IPFD and pancreatic fibrosis are underway, this field of research remains in its nascent stages. It currently suffers from a lack of large-scale clinical validation, and the precise targeted pathways involved in this regulatory network are still unclear, necessitating further in-depth investigation.

## Conclusion and Outlook

AP represents a prevalent digestive system emergency characterized by pancreatic tissue edema, inflammatory infiltration, hemorrhage, and necrosis. Certain cases may progress to SAP, which is accompanied by life-threatening complications including SIRS and MODS, significantly increasing mortality. In recent years, with the increasing prevalence of obesity and metabolic syndrome, IPFD, as a pancreatic pathological lesion closely associated with lipid metabolism disorders, has exhibited a progressive expansion of its affected population. Clinical studies have proven that IPFD plays an important role in the occurrence and progression to AP, while specific mechanisms remain unclear. Based on current available evidence-based medical evidence, this article reviews the mechanisms by which IPFD regulates the progression to AP, with the aim of offering novel insights and a theoretical foundation for inhibiting this pathogenic process.

Current evidence indicates that IPFD drives the progression of AP by modulating the pancreatic microenvironment through multiple pathophysiological mechanisms. First, the primary pathogenic mechanism of IPFD involves the hydrolysis of substantial amounts of FFAs from adipose tissue, leading to lipotoxicity. FFAs are transported into acinar cells via FATP and FABP. This transport induces calcium overload, damages mitochondria and the endoplasmic reticulum, and initiates necroptosis. Furthermore, intracellular FFAs undergo β-oxidation in mitochondria, producing substantial ROS that aggravate inflammatory necrosis and ferroptosis. In vascular endothelial cells, FFAs bind to membrane-surface CD36, induce oxidative stress, compromise endothelial integrity, and trigger intense vasoconstriction. This ultimately results in pancreatic microcirculatory disturbances that impair normal cellular metabolism and function. In ductal cells, FFAs not only bind to CFTR to induce calcium overload and interfere with cell metabolism but also interfere with the absorption of fat-soluble vitamins, inducing the formation of protein clots in the duct. These effects ultimately lead to pancreatic juice excretion disorders and inappropriate pancreatic enzyme activation, exacerbating pancreatic autodigestion. Second, IPFD accelerates the activation of pancreatic enzymes through physical and chemical pathways and forms a vicious cycle. Essentially, IPFD is characterized as a diffuse phenomenon of fat accumulation within pancreatic tissue. Adipose tissue compresses the pancreatic duct, which increases the difficulty of pancreatic juice excretion, elicits inflammatory responses, modulates immune cell reactivity, and promotes trypsinogen activation, thereby driving AP toward greater severity. Moreover, activated pancreatic lipase can hydrolyze IPFD, releasing large amounts of FFAs and creating a self-perpetuating cycle of “lipotoxicity-impaired pancreatic juice excretion-inappropriate enzyme activation”. Third, IPFD-mediated endocrine dysfunction amplifies the inflammatory response. Lipid metabolism disorders associated with IPFD contribute to IR, which activates the NF-κB signaling pathway and suppresses the expression of anti-inflammatory factors 118. Meanwhile, IPFD stimulates glucagon secretion, creating a hyperglycemic microenvironment that activates both the Notch signaling pathway and the NLRP3 inflammasome, which together amplify the inflammatory cascade. Fourth, IPFD disrupts the balance between pancreatic tissue damage and repair. Excessive fat deposition between pancreatic lobules recruits substantial numbers of CCR2⁺ macrophages, which replace fibroblasts and weaken the physical barrier normally formed by fibrous connective tissue. Emerging evidence also suggests that FFAs produced by lipolysis of IPFD induce pancreatic tissue fibrosis, which establishes a conducive microenvironment for the infiltration and pathological proliferation of pancreatic adipocytes and ultimately leads to pancreatic functional impairment.

Although the mechanisms through which IPFD regulates the pancreatic microenvironment to exacerbate progression of acute pancreatitis have been extensively studied, many critical issues still require urgent resolution. First, a standardized definition and precise quantification of IPFD remain to be unified. While many scholars recognize the significant impact of IPFD on digestive, endocrine, and neoplastic diseases, the condition is still described by multiple synonymous professional terms. The indiscriminate use of these concepts may compromise the comparability of data across different studies. Currently, invasive pancreatic tissue biopsy is regarded as the gold standard for measuring IPFD. However, this procedure entails a significant risk of complications, which limits its clinical applicability 6. In clinical practice, ultrasound, computed tomography (CT), and magnetic resonance imaging (MRI) are commonly employed for assessment, yet accurate quantification of IPFD remains challenging due to the retroperitoneal location of the pancreas and the surrounding adipose tissue. Second, the regulatory network through which IPFD exacerbates AP via multiple pathways remains incompletely unclear. This review elaborates on the progression of AP from four perspectives: IPFD-induced damage via lipotoxicity, promotion of premature pancreatic enzyme activation leading to a vicious cycle, disruption of endocrine regulation that amplifies inflammation, and inhibition of pancreatic tissue fibrosis dysfunction that amplifies pancreatic injury. Intersecting regulatory mechanisms and shared targets exist among these pathways. Therefore, further research is necessary to clarify the mutual regulatory networks underlying these mechanisms and to determine whether additive or synergistic effects are present.

## Figures and Tables

**Table 1:** Mechanisms of IPFD-induced endocrine hormone disruption exacerbating AP progression

Secretion source	Endocrine hormone	Hormone level change in IPFD state	Key regulatory mechanism	References
Islet Cells	Insulin	Decreased	Inhibits the NF-κB signaling pathway and reduces the inflammatory response	52
			Activates the STAT3 signaling pathway and enhances the anti-inflammatory effect	53
	Glucagon	Increased	Activates Notch signaling pathway, regulates macrophage polarization to M1 phenotype	63
			Activates NLRP3 inflammasome	64
Adipocytes	Leptin	Increased	Recruits peripheral blood monocytes and tissue macrophages	42, 43
			Induces TNF-α expression, activates intracellular MAPK signaling pathway	44
	Adiponectin	Decreased	Activates AMPK signaling pathway, inhibits lipid peroxidation and inflammatory response	28, 69
Gastric X/A Cells	Ghrelin	Increased	Induces pancreatic enzyme activation and exacerbates pancreatic autodigestion	75-78
